# A Machine Learning Approach Investigating Consumers’ Familiarity with and Involvement in the Just Noticeable Color Difference and Cured Color Characterization Scale

**DOI:** 10.3390/foods12244426

**Published:** 2023-12-10

**Authors:** Guillermo Ripoll, Begoña Panea, María Ángeles Latorre

**Affiliations:** 1 Animal Science Department, Centro de Investigación y Tecnología Agroalimentaria de Aragón (CITA), Avda. Montañana 930, 50059 Zaragoza, Spain; gripoll@aragon.es; 2 Agrifood Institute of Aragon-IA2, CITA-University of Zaragoza, Avda. Miguel Servet, 177, 50013 Zaragoza, Spain; malatorr@unizar.es; 3 Facultad de Veterinaria, Universidad de Zaragoza, Avda. Miguel Servet, 177, 50013 Zaragoza, Spain

**Keywords:** just-noticeable, difference, JND, JNCD, delta E, consumer, machine learning

## Abstract

The aim of this study was to elucidate the relations between the visual color perception and the instrumental color of dry-cured ham, with a specific focus on determining the Just Noticeable Color Difference (JNCD). Additionally, we studied the influence of consumer involvement and familiarity on color-related associations and JNCD. Slices of ham were examined to determine their instrumental color and photos were taken. Consumers were surveyed about color scoring and matching of the pictures; they were also asked about their involvement in food, familiarity with cured ham, and sociodemographic characteristics. Consumers were clustered according to their level of involvement and the JNCD was calculated for the clusters. An interpretable machine learning algorithm was used to relate the visual appraisal to the instrumental color. A JNCD of $\Delta E_{ab}^{*}$ = 6.2 was established, although it was lower for younger people. $\Delta E_{ab}^{*}$ was also influenced by the involvement of consumers. The machine-learning algorithm results were better than those obtained via multiple linear regressions when consumers’ psychographic characteristics were included. The most important color variables of the algorithm were L* and $h_{ab}$. The findings of this research underscore the impact of consumers’ involvement and familiarity with dry-cured ham on their perception of color.

## 1. Introduction

For decades, dry-cured hams have been sold as the whole hind pig leg, but there is an increasing trend of consuming packets of sliced ham. According to data published by the Spanish Government [1], sales dry-cured ham commercialized as packed sliced ham increased from 41.9% in 2008 to 52.5% in 2013 and to 61.2% in 2020. In this purchase scenario, consumers have the chance to consider several product aspects, and color plays a major role in consumer decisions [2,3]. Many studies on meat and meat products have related the *tristimulus* coordinates of the CIELab color space to hue angle and chroma, or to other indicators like b*/a* or the 630 nm and 580 nm reflectance ratios to consumers’ color perception, or even their purchase intention [4,5,6,7]. However, the aim of relating color perception to instrumental color is not trivial. First, the relation of trichromatic coordinates to color visual appraisal is not linear [4,5]. In addition, defining color exclusively by L*, a* and b* separately can lead to misleading interpretations. It is recommend to define colors by luminosity, saturation, and hue angle [4,8] because these factors are more easily interpreted by the human eye [9,10]. Moreover, the b* coordinate (blue and yellow) is not intuitively associated with meat color [2,11] and its use is complicated for meat evaluators [12].

Apart from the relation between instrumental color and perceived color, the difference in color $\left(\Delta E_{ab}^{*}\right)$ between two *stimuli* must be considered. The Just Noticeable Color Difference (JNCD) is defined as the $\Delta E_{ab}^{*}$ between two color *stimuli* reported as different by 50% of people [13]. Theoretically, the minimum average $\Delta E_{ab}^{*}$ distinguishable by the human eye has been reported to be 2.2 [14,15]. However, the JNCD depends on factors related to *stimuli* (spatial and temporal distance, size, etc. There are no studies that describe the JNCD of dry-cured ham and very few that apply the JNCD to meat or meat products [4,16,17]. However, the color of dry-cured ham is one of the most important cues at the purchase time [2,3], especially when the dry cured ham is sold sliced. Therefore, the knowledge of the JNCD of a food is essential to develop a control quality guarantees the uniformity of the product.

Finally, perception is a dynamic concept that incorporates complex consumer behavior aspects, such as learning, motivational and contextual factors [18]. Therefore, perception of color and color matching differ between genders, age groups and nationalities [5,7,15]. In this sense, people older than 60 years perceive colored surfaces to be less vivid than subjects aged under 30 years [19]. In addition to sociodemographic characteristics, the psychographic characteristics of consumers also affect color perception and color matching. Indeed, light-colored lamb meat is more highly valued by traditional consumers, showing that the color of the meat can determine purchase intention [3]. Other authors have observed differences in color acceptability according to age; older consumers generally have more experience in formulating color opinions [5]. Consumers’ familiarity and degree of experience with a product refer to their understanding of the product and their ability to evaluate its quality [20]. Familiarity with the product and consumer attitudes can modify food acceptability [21,22]. Other authors report that consumers’ self-confidence, social aspirations, and personal ideologies influence responses to visual *stimuli* [23].

Another interesting dimension of consumers is involvement in food. Involvement is related to how relevant consumers perceive a product to be in line with their personal needs, values, interests, ego, and motivation for a given situation [24,25]. Consumer involvement is defined as a state of energy (arousal) that a person experiences in relation to consumption-related activity [26]. Other authors understand it as the degree to which a product is centrally related to a consumer’s value system and refer to feelings of interest in, concern for, and enthusiasm about products [27]. Very involved consumers can pay attention to specific sensory inputs because they are motivated to spend the time and effort required to understand a product’s nature. Hence, only very experienced and dedicated respondents (measured in terms of involvement, innovativeness, and knowledge) seem to perceive samples’ similarities/dissimilarities with subtle sensory differences [28]. Fortunately, web surveys provide valid standardized and ambitious (in terms of number of respondents) means of accessing different consumer profiles and asking them about the color acceptance of meat [5,6,29].

Given the complex and nonlinear relations between instrumental and perceived color, machine learning algorithms can be a useful tool for transcribing unstructured knowledge; it is relatively accessible to convert this knowledge into a set of instructions, and thus, into structured knowledge [30]. In the past few years, different nonlinear methods such as machine learning algorithms have been used to address problems in which linear methods have not provided the expected results [4,30,31,32,33]. The Cubist algorithm demonstrates a better performance than other machine learning algorithms like XGboost or Random Forest to solve problems in diverse areas [34,35]. Besides, Cubist does not act as a “black box” like other algorithms do and it is highly interpretable. This high interpretability is a marked advantage, allowing the data structure to be identified outside of prediction or classification. In the present manuscript, we argue that consumers perform their visual appraisals of meat by means of knowledge that can be computationally schematized. This approach has been applied before to beef and kid goat meat colors, and has demonstrated that the relation between CIEL*a*b* color variables and how humans perceive of meat color by humans is not linear [4,33]. This experiment represents a novelty that allows a relationship between instrumental color and visual evaluation of dry-cured ham using nonlinear approaches such as machine learning tools, since there are no studies focused on these relationships that are far from linear correlations or linear regressions. There are some papers dealing with nonlinear relations but in other meat products [4,33].

Considering the previous context, we hypothesize that there are different JNCDs for every group of consumers according to their involvement with food, familiarity with cured ham, and other sociodemographic characteristics. The second hypothesis of the study is that the relationship between visual appraisal of the color of the cured ham with the instrumental color is nonlinear, and this relationship can be unveiled using machine learning algorithms. Finally, it is also hypothesized that these nonlinear relationships depend on the sociodemographic and psychographic characteristics of consumers.

## 2. Materials and Methods

### 2.1. Experimental Samples

**Figure 1:**
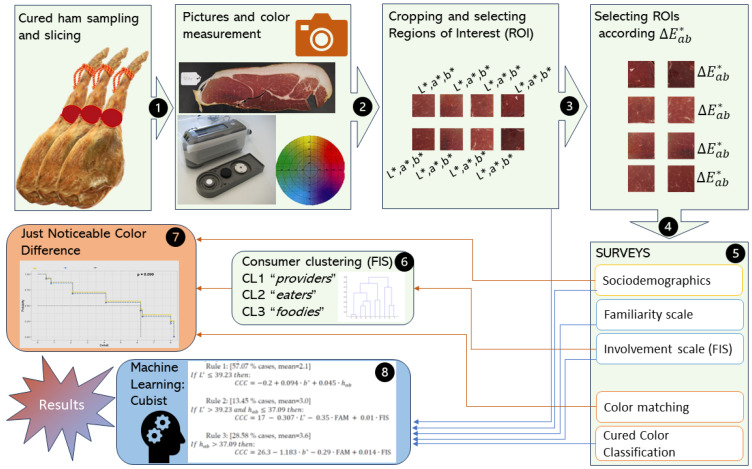
Scheme of the experimental flow from the sampling of hams (1), following the data collection through measurement of instrumental color (2, 3 and 4) and the online surveys completed by consumers (5) until the clustering of consumers according to the involvement scale (6), the estimation of the Just noticeable Color Difference according to the characteristics of consumers (7), and the relationship between the visual appraisal of consumers using the Cured Color Classification and characteristics of consumers. Red lines are the inputs of steps (6) and (7), and blue lines are the inputs of the (8)th step.

Ten dry-cured hams from Duroc × (Landrace × Large White) gilts (5 non-castrated gilts, 5 immunocastrated gilts) intended for the Spanish Protected Designation of Origin (PDO) “Teruel ham” [35] were used in this trial. All the animals (5 entire gilts and 5 immunocastrated gilts) received the same management and feeding were slaughtered on the same day (average body weight of 133 kg) in the same slaughterhouse. For more information about animals, please see Pérez-Ciria et al. [36]. Ham was taken from the left-hand side of each carcass and trimmed. Next, all the pieces were taken to the cellar to be dry-cured according to PDO “Teruel ham2 specifications [37]. The dry-curing process lasted from 19.7 to 20.2 months. Once completed, the dry-cured hams were boned and transversally cut at the coxofemoral joint level. Then, a 2 mm thick slice was cut from each ham using a Sammic GC-300 slicer machine (Sammic SL, Azkoitia, Spain). The visible muscles of the slices were *biceps femoris*, *semimembranosus* and *quadriceps femoris*. The experimental process is described in Figure 1.

### 2.2. Capturing Images of Chops

One slice per ham was placed in a Cubelite Lastolite portable studio (Vitec Imaging Distribution UK, Leicestershire, UK) on a black background. The studio was placed beneath two lightbulbs with five Cromalite bulbs (Cromalite S.L, Barcelona, Spain). Each lightbulb was characterized by 28 W, 5200 K and 1600 lm, which delivered 1500–1600 lx to the sample surface. An Olympus Pen E-PL1 12.3 Mpx camera with a M.Zuiko digital 14–42 mm 1:3.5–5.6 L lens (Olympus Imaging Corp., Shinjuku-ku, Tokyo) was placed over the sample with a tripod. The camera’s exposure settings were manually adjusted to an ISO of 400, a lens aperture of F5.6, and a shutter speed of 1:125. Photos of the slices were taken, and the images were saved in the raw format; no flash, filters or other lenses were applied. No other image editing or processing of manipulation was applied other than the cropping of images.

### 2.3. Color Measurements

**Figure 2:**
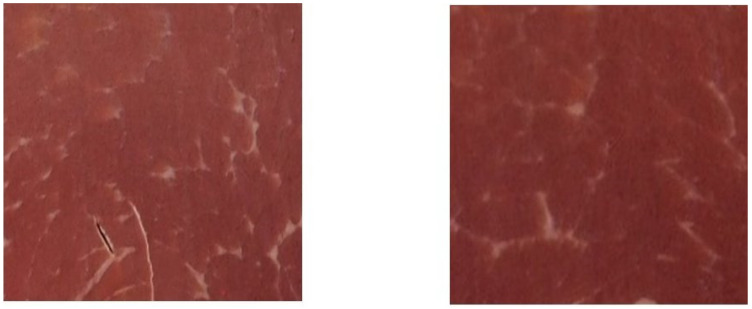
Pair of region of interest (ROI) of *biceps femoris* muscle corresponding with a $\Delta E_{ab}^{*}$ = 8.0. ROIs are squares of 3 × 3 cm with a 1.5-width white background between them.

The color of three muscles from the same slices of ham (*biceps femoris*, *semimembranosus and quadriceps femoris*) was measured by avoiding excessive marbling areas. Muscle colors were measured at 0% UV and an observer angle of 10°, with zero and white calibration, using a Minolta CM-600d spectrophotometer (Konica Minolta Holdings, Inc., Osaka, Japan) in the CIE L*a*b* 1986 space with the specular component included. The measurement area (diameter of 8 mm) was covered with a CM-A183 glass protected mask (Konica Minolta Holdings, Inc., Osaka, Japan). The employed illuminant was D65. The lightness (L*), redness (a*) and yellowness (b*) indexes were recorded with the SpectraMagic NX software (Minolta Co., Ltd., Osaka, Japan), and hue angle $\left[h_{ab}=\tan^{-1}\left(\frac{b*}{a*}\right)\cdot \frac{180}{\pi }\right]$ and chroma $\left[C_{ab}^{*}=\sqrt[{}]{{\left(a*\right)}^{2}+{\left(b*\right)}^{2}}\right]$ were calculated. Color measurements were repeated 3 times for each sample, and the spectrophotometer was rotated on the horizontal plane before each measurement. The mean of the three readings was used for the statistical analysis. The differences in color among the 30 collected readings (10 slices × 3 muscles) were calculated as $\Delta E_{ab}^{*}=\sqrt{{\left(\Delta L*\right)}^{2}+{\;\left(\Delta a*\right)}^{2}+{\left(\Delta b*\right)}^{2}}$. Then, the pairs of the same muscles covering color differences from 0 to 9 were selected. A region of interest (ROI) of 3 × 3 cm was cropped from each picture of muscles to be used for the online survey. The selected pairs of ROIs were placed together at a distance of 1.5 cm on a white background (Figure 2) and utilized for surveys for color scoring and matching purposes.

### 2.4. Survey Design and Dissemination

Two surveys were designed for the current study. The geographical scope of surveys was restricted to Spain to rule out responses from consumers from other countries. The study was conducted using Google Forms (Google, LLC, Los Angeles, CA, USA) and survey data were input into an MS Excel sheet. Questionnaires were tested by researchers who may or may not have been involved in the study and were adapted until the final version was approved. A compromise had to be reached for thoroughness, simplicity, and shortness. Questionnaires were distributed using social media websites. According to the population of Spain (48 million people), a confidence level of 99%, and an error margin of 4.4, a minimum of 860 valid surveys was needed. A divulgation message was included to ask the receivers to further broadcast the web-link to other people on their respective electronic mailing lists and in their social networks. For those receivers willing to participate in the survey, the survey welcome page was immediately accessible after they clicked on the web link. The questionnaire was anonymous to guarantee higher participation and honesty levels. Personal data, such as identification or electronic mail address, were not required, and there was no financial compensation. The participants were clearly informed about the study’s aim and gave implicit consent for the information they supplied in this research work to be used according to European regulations [38]. This study was conducted according to the Declaration of Helsinki for studies on human subjects.

The surveys were arranged in three categories: (a) sociodemographic; (b) psychographic; (c) visual appraisal of ROIs. Both surveys contained sections (a) and (b) but differed in the pictures included in section (c). The information collected in section (a) included gender, age, living environment, and level of education. The information acquired in section (b) included consumers’ involvement with food and their familiarity with dry-cured ham. The scale used to assess consumer involvement was the Food Involvement Scale (FIS) [25]. The participants rated their level of agreement with 12 items on a 7-point scale with labeled endpoints (disagree strongly, agree strongly). The items were: (1) I do not think much about food every day; (2) cooking or barbequing is not much fun; (3) talking about what I ate or am going to eat is something I like to do; (4) compared with other daily decisions, my food choices are not very important; (5) when I travel, one of the things I anticipate most is eating the food there; (6) I do most or all of the clean up after eating; (7) I enjoy cooking for others and myself; (8) when I eat out, I do not think or talk much about how the food taste; (9) I do not like to mix or chop food; (10) I do most or all of my own food shopping; (11) I do not wash dishes or clean the table; (12). I care whether or not a table is nicely set. To score the scale, item numbers 1, 2, 4, 8, 9 and 11 were reversed. Once reversed, the scores for all 12 items had to be summed for each individual, which gave the total FIS score. Therefore, the FIS scale had a theoretical range of 12–84 and a mid-point of 48. In addition, two subscales were defined as follows: items 6, 11 and 12 were added together to make up one subscale, referred to as the “Set and Disposal” (S&D) involvement subscale. This subscale has a theoretical range of 3–21 and a middle point of 12. All other items should be added together to make up the other subscale, referred to as the “Preparation and Eating” (P&E) involvement subscale. This subscale had a theoretical range of 9–63 and a mid-point of 36. The familiarity level was assessed using the scale proposed by Backstrom et al. [39]. The familiarity scale consisted of five options: (1) I do not recognize the product; (2) I recognize the product, but I have not tasted it; (3) I have tasted the product, but I do not use it; (4) I occasionally eat the product; (5) I regularly eat the product. Finally, section (c) in each survey included five pairs of ROIs that resulted in the following color differences: 0.8, 2.1, 4.1, 6.3 and 8.0 for survey A and 0.5, 2.1, 4.1, 6.2 and 8.2 for survey B. Pairs of ROIs were randomly presented in the survey and the respondents were asked if the color of both ROIs was the same. The respondents also assessed the color of each ROI using the Cured Color Characterization (CCC) proposed by AMSA [40]. The CCC scale consisted of 8 options: (1) very dark red cured color; (2) moderately dark red cured color; (3) slightly dark red cured color; (4) reddish-pink cured color; (5) pinkish-red cured color; (6) slight pinkish-red cured color; (7) pinkish cured color; (8) light pinkish cured color.

### 2.5. Statistical Analysis

All the data analyses were performed in the R programming environment v.3.6.3 [41].

#### 2.5.1. Consumer Clustering According Food Involvement Scale

A factor analysis using principal components as the extraction method was performed with the 12 FIS questions to reduce the number of dimensions. The Kaiser–Meyer–Olkin factor adequacy was 0.80, which indicates that the different items measure one single construct and therefore may be aggregated. The individual co-ordinates of the observations to the 3 main dimensions (with eigenvalues > 1) obtained from the factor analysis were retained and used in the cluster analysis. A hierarchical cluster analysis (using Ward’s method for aggregation and Euclidian distance) was run to identify homogeneous groups of respondents according to the involvement profile (cluster). The differences between the involvement groups relating to the FIS questions and FIS subscales were assessed by a one-way analysis of variance (ANOVA) with the involvement profile as the fixed effect and the FIS questions and FIS subscales as dependent variables. The Duncan test was used to compare means, and the level of significance was *p* < 0.05. 

Dependence between involvement profiles and nominal variables (gender, age, environmental living, level of education and familiarity with dry-cured ham) was studied with the χ^2^ test. To interpret the patterns of association between the studied variables, the corrected standardized residual between the observed and expected cases in each cell bigger than |1.96| was considered.

#### 2.5.2. Estimation of JNCD

To determine the JNCD, a Kaplan–Meier survival analysis was conducted using the *survminer* [42] and *survival* [43] R packages. The estimate of JNCD was the median; that is, the $\Delta E_{ab}^{*}$ between two color *stimuli* (ROI) which is noticeable by 50% of consumers. The log-rank statistic was computed to determine if there was a significant difference in the survival curves between the involvement profiles, genders, age, environmental living, level of education, and familiarity with dry-cured ham. Significant differences were considered if the *p*-value was less than 0.05.

#### 2.5.3. Relationship between Visual Appraisal and Instrumental Color

Two multivariate linear regressions (MLR) models were developed using the *lm* function. The first MLR model was carried out with the CCC as the dependent variable and the instrumental color variables were the independent variables. The second MLR model was carried out with the CCC as the dependent variable and the instrumental color variables and FIS and familiarity were the independent variables.

The *Cubist* package was used to develop rule-based predictive models with the machine learning algorithm Cubist [44]. This algorithm employs input data (instrumental color variables, FIS and familiarity) to generate a decision tree with linear functions in all leaves to predict the CCC. The coefficient of determination (R^2^) measures the agreement between the cases’ actual values for the target attribute and those values predicted by the model [44]. While decision tree algorithms (such as Cubist) may have slightly lower accuracy than other ML models, their high interpretability has great advantages because a major goal of our work is to gain insights into the instrumental color variables that explain the visual appraisal of consumers. Due to the aim of the interpretability, we decided not to build an ensemble of trees or use other ML methods like Random Forest which act like “black boxes”. The hyperparameters of Cubist were tuned to increase the predictive power using the Residual Standard Error as a criterion.

Two ML model were developed using Cubist. The first ML model was carried out with the CCC as the dependent variable and the instrumental color variables were the independent variables. The second ML model was carried out with the CCC as the dependent variable and the instrumental color variables and FIS and familiarity were the independent variables.

## 3. Results

### 3.1. People and Dry-Cured Ham Characterization

The survey was completed by 877 consumers, of which 867 responses were considered valid. The number of valid responses ensures a confidence level of 99% and a margin error of below 5%. The respondents were 59.2% woman and 40.8% men. In total, 5% people were below 20 years of age, 32% were between 20 and 29 years, 16% were between 30 and 39 years, 19% were between 40 and 49 years, 20% were between 50 and 59 years, and 8% were older than 59 years. Regarding the environmental level, 65% of the people lived in cities, 10% in big towns, 5% in medium-sized towns, and 20% in small towns. Their level of education was distributed as follows: 65% had completed high-level studies (university, Ph.D.…), 30% medium-level studies (high school) and 5% low-level studies or no studies.

**Figure 3:**
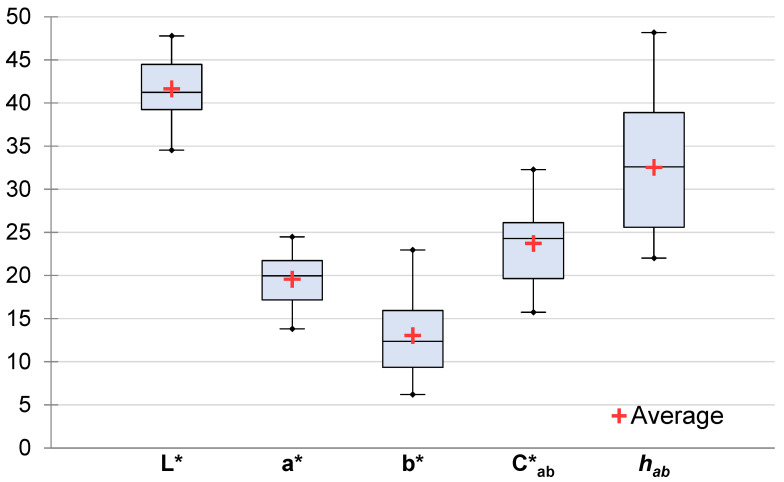
Statistics of instrumental color of dry-cured ham. The box of a boxplot starts in the first quartile (25%) and ends in the third (75%). The black line inside the box represents the median and the red cross represent the average. The segments on each side of the box extend to one and a half times the interquartile range. L* = lightness, 0 to100. a* = redness index and b* = yellowness index are unbound, usual limits −128 to +128. $C_{ab}^{*}$ = Chroma; $h_{ab}$= hue angle.

Dry-cured ham color is shown in Figure 3. The average of L*, a*, b*, $C_{ab}^{*}$ and $h_{ab}$ was 41.66, 19.57, 13.05, 23.74, and 32.55, respectively. The standard deviation of L*, a*, b*, $C_{ab}^{*}$ and $h_{ab}$ was 3.301, 2.783, 4.943, 4.660, and 7.758, respectively.

### 3.2. Food Involvement Profiles

**Figure 4:**
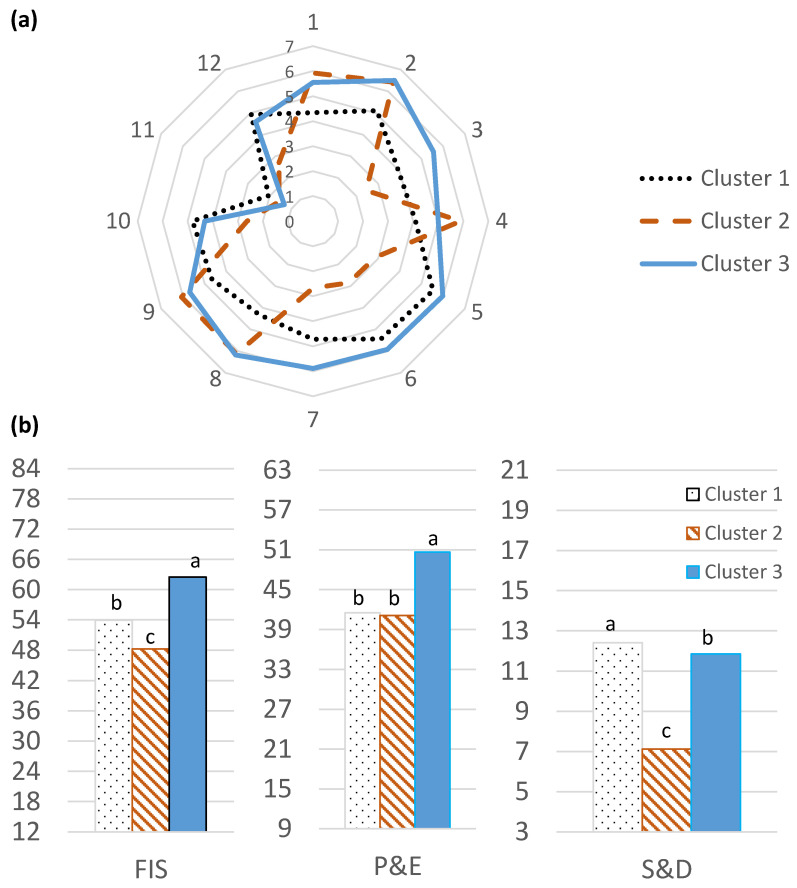
(**a**,**b**) Differences between involvement profiles of consumers. Bars with different letters (a,b,c) means significant differences at *p* < 0.05 level. FIS scale: 1. I do not think much about food each day; 2. Cooking or barbequing is not much fun; 3. Talking about what I ate or am going to eat is something I like to do; 4. Compared with other daily decisions, my food choices are not very important; 5. When I travel, one of the things I anticipate most is eating the food there; 6. I do most or all of the clean up after eating; 7. I enjoy cooking for others and myself; 8. When I eat out, I don’t think or talk much about how the food taste; 9. I do not like to mix or chop food; 10. I do most or all of my own food shopping; 11. I do not wash dishes or clean the table; 12. I care whether or not a table is nicely set. Item reversely scaled. Seven-point Likert scales, ranging from 1 = “disagree strongly” to 7 = “agree strongly”. The scale of each figure is adjusted to the scale used for FIS, P&E and S&D and the red line is the theoretical middle point of scale. Cluster 1 participants are called *providers*, Cluster 2 participants are called *eaters,* and Cluster 3 participants are called *foodies*.

Two statistical approaches were investigated to cluster the respondents into food involvement profiles. The PCA, together with hierarchical clustering, optimized interclasses variation, but differences between clusters on some scale items and FIS subscales were missing. The use of hierarchical clustering with all the scale items reported slightly narrower interclasses variation, but differences in the scale items and subscales were maximized. Therefore, the direct use of hierarchical clustering was chosen because the definition of clusters was more efficient. The cluster effect on the individual items of the FIS and FIS subscales was significant (*p* < 0.0001). The means are shown in Figure 4. In general, the three clusters were moderately involved with food. Cluster 2 had FIS values in the middle of the scale (48.2). Cluster 1 was higher (*p* < 0.05), with values of 53.9. Cluster 3 was the most involved and had the highest values of 62.5 (*p* < 0.05). The three clusters had higher P&E subscale scores than the mid-point (36), which showed a high preference for preparing and eating. Clusters 1 and 2 were similar (*p* < 0.05) but differed from Cluster 3 with the highest score (50.6). Regarding the S&D subscale, Clusters 1 (12.4) and 3 (11.8) had values around the middle of the scale, but they were significantly different (*p* < 0.05). Cluster 2 had a lower score (7.1) (*p* < 0.05) and indicated very little involvement in the attitudes to serving and disposal. 

No relation appeared between the clusters and gender (χ^2^ = 0.967; *p* = 0.6), living environment (χ^2^ = 9.230; *p* = 0.3), and familiarity with dry-cured-ham (χ^2^ = 9.180; *p* = 0.3), but a significant association appeared between clusters and age (χ^2^ = 30.990; *p* < 0.001) and level of education (χ^2^ = 27.235; *p* < 0.0001). Cluster 1 (50.9%) included people of all ages and with all the levels of education who usually do food shopping, wash dishes, clean table, and care whether the table is nicely set, but attach less importance to cooking or eating activities. This profile could be called *providers*. Cluster 2 (24.2%) mainly contained people older than 59 years, with only a few people younger than 20 years, who are not currently studying, who simply enjoy eating, but do not buy food, cook, wash dishes, clean the table, or look after setting the table. The profile of this group of respondents could be defined as *eaters*. Finally, Cluster 3 (24.9%) comprised respondents who were mostly younger than 20 years with a high level of education, who could be named *foodies* because they are involved in most of the eating activity steps, from thinking about eating to cooking, eating, and cleaning, but do not like washing up.

### 3.3. Just-Noticeable Color Difference

**Figure 5:**
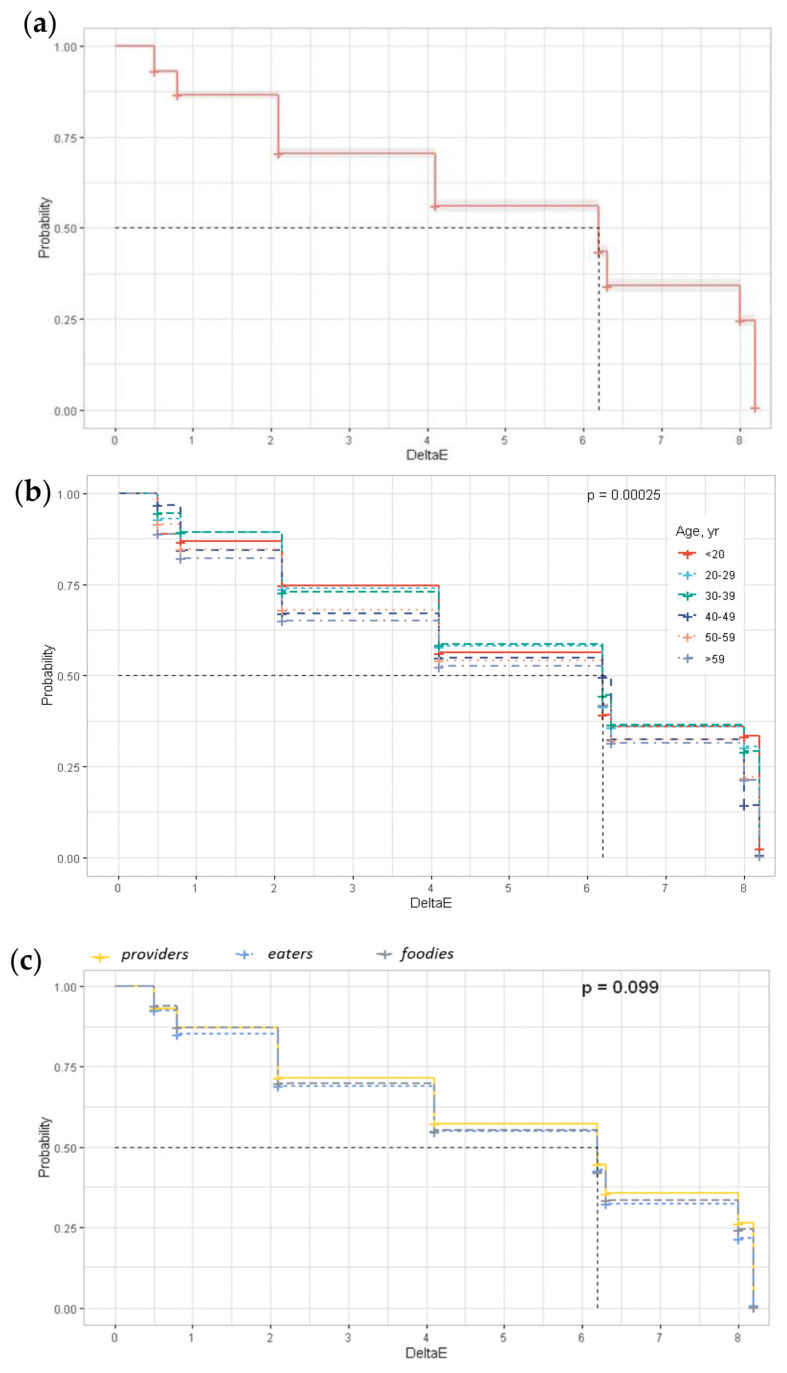
Kaplan–Meier survival curves (**a**) all data, (**b**) per age, (**c**) per cluster.

The Kaplan–Meier survival curves employed to determine the JNCD are shown in Figure 5. There was no effect for gender (*p* = 0.2), level of education (*p* = 0.7), familiarity (*p* = 0.4) and cluster (*p* = 0.099) on the JNCD. However, the JCND was affected by age (*p* = 0.0003). The JNCD (50%) for the sample corresponded to a $\Delta E_{ab}^{*}$ = 6.2 ± 0.0084, while the JNCD (25%) was 2.1 and the JNCD (75%) was 8.0. The relation between the JNCD and percentiles was adjusted to a second-degree polynomial curve with R^2^ = 0.98 (JND = −0.0007x^2^ + 0.1612x − 0.6716) where x was the percentile of people who detected color differences. Differences due to age were more evident within the $\Delta E_{ab}^{*}$ range between 2.1 and 4.1. In general terms, more people younger than 39 years began to notice different *stimuli* with $\Delta E_{ab}^{*}$ = 2.1 than people older than 39 years. Regarding the clusters, it would seem that *providers* noticed differences before the other consumer groups.

### 3.4. Relationship between Instrumental Color and Visual Appraisal

**Figure 6:**
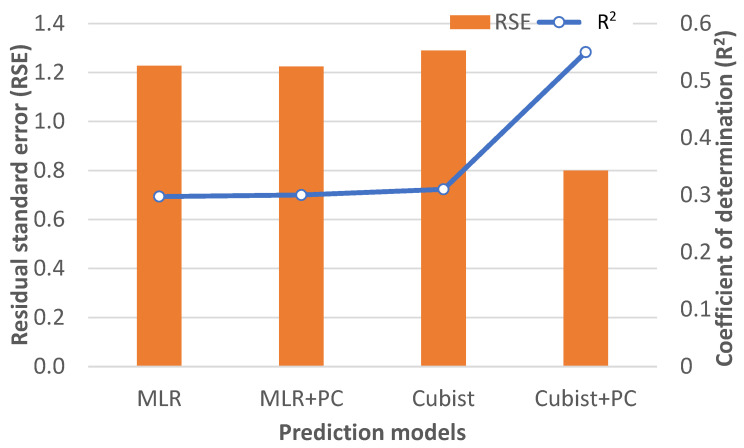
Coefficient of determination (R^2^) and residual standard error (RSE) of the multiple linear regressions (MLR) and Cubist machine learning algorithm with or without psychographic characteristics (PC) relating instrumental color to Cured Color Classification.

Four models were studied, mainly multiple linear regression (MLR) and the machine learning algorithm Cubist, with or without the inclusion of two psychographic characteristics (PC) (Food Involvement Scale-FIS and familiarity-FAM) to estimate the Cured Color Classification. The residual standard error (RSE) and coefficient of determination (R^2^) of each model are shown in Figure 6. MLR and MRL + PC performed similarly with RSE = 1.23 and R^2^ = 0.30. Cubist without PC provided a complex prediction tree with seven rules, but RSE and R^2^ were not much better than MLR models. However, when PC were included, the prediction tree was simplified to three rules with an R^2^ = 0.55 and an RSE = 0.80. The proposed prediction tree of Cured Color Classification (CCC) was

Rule 1: [57.07% cases, mean = 2.1]

If $L^{*}\leq 39.23$, then:

$$CCC=-0.2+0.094\cdot b^{*}+0.045\cdot h_{ab}$$

Rule 2: [13.45% cases, mean = 3.0]

If $L^{*}>39.23\;and\;h_{ab}\leq 37.09$, then:

$$CCC=17-0.307\cdot L^{*}-0.35\cdot FAM+0.01\cdot FIS$$

Rule 3: [28.58% cases, mean = 3.6]

If $h_{ab}>37.09$, then:

$$CCC=26.3-1.183\cdot b^{*}-0.29\cdot FAM+0.014\cdot FIS$$

Rules were mainly based on L* and $h_{ab}$, while regressions used b*, L*, $h_{ab}$, *FAM* and *FIS*.

## 4. Discussion

### 4.1. Dry-Cured Ham Color

The reported instrumental dry-cured ham color covers a wide range of values because it primarily depends on the measured muscle. Three muscles were measured in the present study. Color is affected by many factors such as the additives used, packaging, or processing [45]. However, dry-cured ham instrumental color remains quite steady throughout its usual shelf life (6–8 months) [45,46], especially for L* and b* [47]. The reported values are normal for this kind of meat product [45,46,48,49,50,51], although some authors have found slightly different values [47,52].

### 4.2. The JNCD of Dry-Cured Ham

There are no data available about the JNCD of meat and meat products, although some approximations have been published. Carrasco et al. [17] reported that evaluators do not discriminate ovine carcasses with a subcutaneous fat color from different fattening systems with ΔE* = 5.2 because carcasses were not evaluated next to one another. In the same way, Babiker et al. [16] reported that goat and lamb meat with $\Delta E_{ab}^{*}$ = 1.97 are not detected by consumers if they are not shown together. When the respondents of two web surveys were asked about the meat of very light kid goat (*cabrito*) carcasses, differences between rearing systems with $\Delta E_{ab}^{*}$ = 4.0 were detected, but no difference was found with $\Delta E_{ab}^{*}$ = 2.4. This was expected because, hypothetically, when $\Delta E_{ab}^{*}$ is below 2.2, *stimuli* cannot be discriminated differently from one another [15]. Other authors reported that $\Delta_{ab}^{*}$ below one is imperceptible, and differences between one and four may or may not be perceptible [53] for nonfood *stimuli*. Finally, some authors propose scales of thresholds without providing empirical evidence [54,55]. Based on the literature and study results, the JNCD of meat products exceeds four, varying by the type of meat and consumer characteristics. Increasing the knowledge of the JNCD of meat and meat products is important in food science experiments to evaluate the importance consumers attach to statistical results. However, defining a visually detectable threshold is difficult because it largely depends on many factors. One factor is the physicotemporal distance between *stimuli* [8], and the closer *stimuli* are, the lower the JNCD is. Color itself is another determinant factor. Hence, the JNCD depends on where two compared colors are located in the color space (e.g., CIELab) [56] because, according to Weber’s law, “the minimum increase of stimulus which will produce a perceptible increase of sensation is proportional to the pre-existent stimulus” [57]. The visual discrimination of $\Delta E_{ab}^{*}$ depends on which color attribute differs (L*, a*, b*, etc.) [58]. Experiments with $\Delta E_{ab}^{*}$ due to isolated trichromatic coordinates are relatively easy to design using printed cards or light bulbs, but it is almost impossible to design such experiments around real meat products. One final question is whether the JNCD varies according to human eye sensitivity. It is widely accepted that women differentiate more colors than males. However, Melgosa et al. [58] showed that gender does not influence the JNCD, which agrees with the results of the present study. In our trial, young people also needed a lower $\Delta E_{ab}^{*}\;$ to discriminate colors than older people, which confirm that color discrimination deteriorates with age [59]. The influence on the JNCD of other consumer characteristics must be evaluated for each meat product. However, these findings are relevant to the meat industry because the industry now uses a threshold in the control of the color of cured ham. This JNCD can be used to implement a pass/fail quality control to ensure homogeneity in the final product.

### 4.3. Relation between Instrumental Color and Visual Appraisal

The ML algorithm that depicts nonlinear relationships between visual appraisal and instrumental color variables achieved better accuracy than MLR models, as it agrees with previous studies [4,33,60]. Although Cubist outperformed MLR, an even greater level of accuracy from a more precise ML model was anticipated. Cubist has hyperparameters that necessitate defining options such as the ensemble of trees and bagging to enhance model accuracy. The utilization of this approach, or the implementation of Random Forest, neural networks, Supported Vector Machines, XGBoost, etc., undoubtedly results in a higher R^2^ and lower RSE. However, these options lack interpretability and act as a “black box”. Thus, using other ML algorithms proves useless in analyzing the underlying links between visual appraisal, instrumental color, and the impact of psychographic traits on consumers.

The obtained results showed that the CCC was based mainly on L* and $h_{ab}$ as thresholds, while the variables used on the linear regressions equations were b*, L*, $h_{ab}$, FAM and FIS. Other authors reported a close relation between visual assessment and *h_ab_*, $C_{ab}^{*}$ and a*/b* rather than to the standalone trichromatic coordinates [4,61,62]. Ripoll et al. [33] also employed the ML algorithm Cubist to relate the visual appraisal and instrumental color of kid goat meat. In agreement with our results, the algorithm used L* as a threshold, and then the main variables included in the linear regressions were $h_{ab}$, b*, $C_{ab}^{*}$ and a*, although $C_{ab}^{*}$ and a* were not included in the algorithm for dry-cured ham. Holman et al. [5] have linearly related, for a given b*, beef color acceptability, while the relation between color acceptability with b* was not linear. Other studies about beef support these results relating visual appraisal to L* rather than to a* [60,63]. Shorthose et al. [64] reported a nonlinear relation of L* to color scores. Albertí et al. [60] indicated an artificial intelligence algorithm to find a function based on L* and *h_ab_* as the most explanatory variables on a scale from 1 (light pink beef) to 5 (dark red beef). The found function selected lightness and hue angle. Based on the literature and results of this study, we recommend that studies on the color of meat and meat products focus on brightness, hue angle, and saturation instead of a* and b* to prevent misunderstandings. Most studies have not considered consumers’ characteristics. However, the present work evidenced that familiarity and involvement affected consumers’ perceptions of dry-cured ham color. Familiarity was positively included in the linear regressions of rules 2 and 3 and resulted in a high CCC. Accordingly, the people who are more familiar with dry-cured ham, such as Spanish consumers vs. Norwegian consumers, better understand quality descriptors [65]. In disagreement with this idea, Holman et al. [5] did not report any effect of red meat consumption on consumers’ visual appraisals. The other studied psychographic characteristic studied was involvement. Lee et al. [66] noted that high product involvement induces a sensitive discrimination of product attributes, which agrees with our results. With sociodemographic characteristics, older people are more sensitive to color cues [67], but they also perceive duller colors than those who are younger [68]. However, the studies of Holman [5,6] did not mention any influence of nationality, gender, or age on the acceptability threshold that they reported. Research indicates that when studying color, it is more advantageous to focus on variables such as lightness and hue angle rather than redness or yellowness. Furthermore, segmentation of ham production based on the final color and psychographic characteristics of the target market can be achieved by considering the impact of familiarity with dry-cured ham and involvement in the food.

## 5. Conclusions

A JNCD for dry-cured ham is established, as 50% of people in our study were able to discriminate between two hams with $\Delta E_{ab}^{*}$ = 6.2, although people younger than 39 years could discriminate hams with a lower $\Delta E_{ab}^{*}$ than older people. The JNCD is slightly affected by consumer involvement. According to the Food Involvement Scale, three consumer groups can be identified: *providers*, *foodies* and *eaters*. Providers are the people who do the food shopping, wash dishes, clean the table and care whether a table is nicely set, but consider cooking or eating to be less important, and discriminate between colors with a lower $\Delta E_{ab}^{*}$ than the other consumer groups. However, this JNCD applies only to dry-cured ham colors. The JNCD must be defined for each meat product and related to consumer characteristics. The clarification of this JNCD could be used to improve quality control in the cured ham industry, where sliced ham is sold in modified atmosphere packaging, even vacuum-packed ham. This JNCD provides the threshold for a pass/fail control, preventing the rejection of ham due to unnoticeable variations in color from the specified standard.

The machine learning algorithm helps us to understand consumers’ visual appraisal, and the results are better than those obtained by multiple linear regressions when including consumers’ psychographic characteristics in the model. The most important color variables of the algorithm are L* and *h_ab_*. Although threshold values of algorithms may differ according to the aperture size of spectrophotometer and other variables, the algorithm provides knowledge about the color cues that consumers use to evaluate dry-cured ham color. Therefore, employing artificial intelligence and nonlinear rules provides a precise approximation of evaluators’ scores and shows that meat color cannot be interpreted linearly or when using CIELab color variables separately. Based on the obtained findings, it can be inferred that the level of consumers’ involvement and previous knowledge regarding dry-cured ham significantly influences their perception of its color. However, it should be noted that the impact on the Just Noticeable Color Difference (JNCD) is only slightly influenced by the involvement. These findings will be useful when devising market strategies aimed at very specific consumer niches.

## Data Availability

The data presented in this study are available on request from the corresponding author. The data are not publicly available due to privacy.
